# The Measurement Invariance of the Student Opinion Scale across English and Non-English Language Learner Students within the Context of Low- and High-Stakes Assessments

**DOI:** 10.3389/fpsyg.2016.01352

**Published:** 2016-09-12

**Authors:** Jason C. Immekus, Dean McGee

**Affiliations:** ^1^Department of Educational Leadership, Evaluation and Organizational Development, College of Education and Human Development, University of Louisville, LouisvilleKY, USA; ^2^Kern High School District, BakersfieldCA, USA

**Keywords:** test-taking motivation, large-scale assessment, measurement invariance, high school students, factor analysis, statistical

## Abstract

Student effort on large-scale assessments has important implications on the interpretation and use of scores to guide decisions. Within the United States, English Language Learners (ELLs) generally are outperformed on large-scale assessments by non-ELLs, prompting research to examine factors associated with test performance. There is a gap in the literature regarding the test-taking motivation of ELLs compared to non-ELLs and whether existing measures have similar psychometric properties across groups. The Student Opinion Scale (SOS; Sundre, 2007) was designed to be administered after completion of a large-scale assessment to operationalize students’ test-taking motivation. Based on data obtained on 5,257 (41.8% ELL) 10th grade students, study purpose was to test the measurement invariance of the SOS across ELLs and non-ELLs based on completion of low- and high-stakes assessments. Preliminary item analyses supported the removal of two SOS items (Items 3 and 7) that resulted in improved internal consistency for each of the two SOS subscales: Importance, Effort. A subsequent multi-sample confirmatory factor analysis (MCFA) supported the measurement invariance of the scale’s two-factor model across language groups, indicating it met *strict factorial invariance* (Meredith, 1993). A follow-up latent means analysis found that ELLs had higher effort on both the low- and high-stakes assessment with a small effect size. Effect size estimates indicated negligible differences on the importance factor. Although the instrument can be expected to function similarly across diverse language groups, which may have direct utility of test users and research into factors associated with large-scale test performance, continued research is recommended. Implications for SOS use in applied and research settings are discussed.

## Introduction

Large-scale assessments are an important accountability tool for student learning that affects K-12 educational practices in the United States (U.S.; Hamilton, 2003). Score-based decisions among policy-makers, educators, school district administrators, and researchers rests on the premise that students put forth their best effort toward test performance. Test-taking effort can be defined as “a student’s engagement and expenditure of energy toward the goal of attaining the highest possible score on the test” (Wise and DeMars, 2005, p. 2). The impact of student effort on test performance indicates that it may be a source of construct irrelevant variance that can affect the reliability and validity of high-stakes assessment scores (Wolf and Smith, 1995; Haladyna and Downing, 2004). This may be reflected in students exerting low effort that results in poor test performance (Haladyna and Downing, 2004; Wise, 2009; Abdelfattah, 2010). Consequently, the validity of resultant scores is questionable due to the fact that they are based on factors extraneous to the measured trait. In the U.S., English Language Learners (ELLs) generally are outperformed on large-scale assessments by non-ELLs. Characteristically, large-scale assessments are considered low-stakes if results have minimum consequences on test-takers, whereas those regarded as high-stakes have direct consequences for examinees (American Educational Research Association [AERA], 2000). For educators and school district administrators seeking to meet the learning needs of a diverse student population, assessing students’ test-taking effort can provide a basis for the extent to which scores can be used for programmatic, evaluative, and accountability purposes.

Large-scale assessments are an integrated component of educational systems. For example, end-of-grade assessments represent low-stakes measures designed to assess students’ attainment of grade standards, where high school exit exams represent high-stakes assessments used by many states as gatekeepers to award students a high school diploma. Depending on assessment type, students’ goal-directed activities may vary due to the consequences attached to test performance (Pintrich and Schunk, 2002). Specifically, test-taking motivation may be less on a low-stakes assessment since one’s performance has little to no implications, whereas motivation may be high on a high school exit exam since performance is tied to the attainment of a high school diploma. Regardless of assessment type, for students to place value on test performance, they must believe that greater effort is worthwhile (Eklöf, 2006). According to Cole et al. (2008), students who place the values of attainment, interest, and usefulness on an assessment will put forth substantially more effort to do well than those ascribe little to no personal value on it. When student test-taking motivation is low, however, it is unclear whether test performance reflects academic proficiency or motivation. Consequently, students’ obtained scores may not represent academic proficiency but instead their test-taking effort (Wise and DeMars, 2010). Wise and Smith (2011) report, “[A] key requirement when evaluating the validity of a test score is that the examinee has devoted sufficient effort to the test items to ensure that the resulting test score accurately reflects the examinee’s actual level of proficiency” (p. 139). The relationship between test-taking motivation and test performance may be a relevant factor to consider when examining test performance disparities across student sub-groups. For example, the persistent lower performance of ELLs on high-stakes assessments warrants consideration of the extent to which their test-taking motivation may be similar to non-ELLs and how it relates to test performance.

Consideration of the test-taking motivation of ELLs is important based on the changing demographics of the U.S. student population and persistence achievement gaps. Specifically, ELLs comprise the fastest growing student population sub-group in the U.S. For example, in 2002–2003, ELLs represented 8.7% of the school-aged population and in 2011–2012 comprised 9.1% (Kena et al., 2014). Academically, ELLs persistently score lower on large-scale assessments than non-ELLs. According to National Assessment of Educational Progress (NAEP), Grade 4 ELLs in 1998 and 2013 scored 43 and 39 points lower than non-ELLs on the English Language Arts (ELA) test, whereas Grade 8 ELLs scored 46 and 45 points lower (Kena et al., 2014). In mathematics, for the years of 1996 and 2013, Grade 4 ELLs scored 24 and 25 points lower than non-ELLs, whereas Grade 8 ELLs scored 46 and 41 points lower, respectively. The extent to which large-scale assessment scores reflect the measured trait absent of extraneous variables is an important issue with direct implications to educational policy and practice to addressing the learning needs of a diverse P-12 student population.

There are a number of factors that affect students’ test performance. Among others, these include student attitude, linguistic complexity of the test items, or noisy testing environment. Particularly among ELLs, language demands of the test items may result in assessment scores reflecting both English language proficiency in addition to the intended measured trait (e.g., mathematics ability). Consequently, language proficiency may be a source of construct irrelevant variance which then limits the construct validity of their scores. Abedi and Gándara (2006) identify the challenges associated with second language acquisition and ensuring fair testing practices for ELLs. The authors identify a range of cognitive and non-cognitive factors associated with ELLs’ performance on large-scale assessments to consider in test development and use. In response, research has examined the effects of test accommodations and modifications on ELL test performance (Abedi et al., 1998, 2001; Abedi and Lord, 2001). Whereas addressing the language complexity in test items is necessary to promote fairness in testing for diverse student groups (Abedi and Lord, 2001; Hansen and Mislevy, 2004), students’ self-beliefs are also a key factor that can affect test performance (Steele, 1997).

High school students may be less inclined to put forth their best efforts when taking large-scale assessments due to a decline in their values and attitudes toward school with age (Otis et al., 2005; Dotter et al., 2009). Bong (2009) indicated that over time student self-perceptions of competence tend to negatively impact academic task engagement and effort. The deterioration of high school students’ motivation may, in part, be attributed to their attitudes toward school and peer influences (Dotter et al., 2009). Among ELLs, personal, schooling, and linguistic factors have been reported to contribute to large-scale test performance (Durán, 2008). While the consequences associated with low and high-stakes assessments may differ for students, results nonetheless have important implications for educational decisions, such as the misclassification of ELL status based on test scores (Abedi, 2008a,b). There is a gap in the literature exists whether ELLs have similar test-taking motivation compared to non-ELLs. Thus, extent to which test-taking motivation may be a factor associated with the interpretation and use of the high-stakes assessment results of ELLs is an important empirical question with direct implications to the validity of large-scale testing programs.

There are several theoretical frameworks that can be used to examine the relationship between students’ test-taking motivation and large-scale assessment performance (e.g., Deci and Ryan, 1985; Bandura, 1986; Ryan and Deci, 2000; Wigfield and Eccles, 2000). One theory that has received attention in the literature on test-taking effort and large-scale assessment performance is the expectancy-value theory (EVT; Eccles et al., 1983; Wigfield, 1994; Wigfield and Eccles, 2000; Eccles and Wigfield, 2002). Within the context of large-scale assessments, EVT posits that test performance is based on the two key elements of expectancy of success and task value (Eccles et al., 1983; Wigfield, 1994; Eccles and Wigfield, 2002). Expectancy of success addresses students’ beliefs about their task performance, whereas task value considers the intrinsic value, usefulness, and cost they assign to the assessment (Eccles et al., 1983; Wigfield and Eccles, 1992, 2000). For instance, EVT posits students’ test-taking effort will varying depending on the assessment type in addition to their perceived value and expectancy of success. Among ELLs, the linguistic complexity of a large-scale assessment may be a deterrent to putting forth a high level of effort to attain a high score. Therefore, the test-taking motivation of ELLs may be an important factor to consider if there is reason to believe it may affect test score reliability and validity.

Expectancy-value theory is well suited to frame investigations into the relationship between test-taking motivation and test performance. For example, the variability of test-taking motivation may include students who give their maximum effort due to high expectancy and value of the assessment to those who exert little to no effort on the assessment (Wise and DeMars, 2005). In other instances, students may initially put forth some effort because test items do not appear to be difficult to answer, but quickly shift to guessing or leaving answers blank because of fatigue or lack of interest (Wise and DeMars, 2005). In high-stakes assessment contexts, for instance, expectancy for success may be greater if the student’s ability beliefs are high or it may be low if the student feels unprepared. For ELLs, challenges associated with second language acquisition and self-beliefs may influence their test-taking motivation. Conversely, a student’s expectancy for success may be negatively affected because of low motivation to perform regardless of ability beliefs. In other words, despite ability, students may not have an expectancy for success because they are not motivated to put forth their best effort. This may be particularly relevant among ELLs in which a host of factors may influence their test-taking motivation on large-scale assessments.

Measures of students’ test-taking effort have been developed to assist with interpreting the validity of large-scale assessment results (Sundre, 1999). One such measure is the Student Opinion Scale (SOS: Sundre, 2007), intended to measure students’ test-taking motivation based on EVT (Eccles et al., 1983; Pintrich, 1989). The 10-item measure is designed to yield scores to operationalize students’ test-taking effort, with higher scores indicative that large-scale assessment scores may be more valid (Sundre, 2007). Whereas scores are not intended to drive decisions about individual students, educators may desire to use the scale to judge the test-taking motivation of student groups. Instrument development and validation was based on data collected across two 4-year universities and a 2-year (Community) college. Investigations of the psychometric properties of scores have found them to be reliable and factor analytic studies have supported its two theoretical subscales: Importance and Effort (Sundre and Finney, 2002; Sundre, 2007). While the psychometric properties of the SOS have been investigated among college student data (Sundre and Kitsantas, 2004; Wise and Kong, 2005; Cole et al., 2008; Thelk et al., 2009; Swerdzewski et al., 2011), additional research is needed on other popuations in which the instrument may serve useful. For example, Thelk et al. (2009) found support for the scale’s two-factor structure among college student data, as compared to a one-factor model. Their findings also found that the SOS two-factor model was invariant (similar) across modes of administration (i.e., computer- based vs. paper-and-pencil testing) and gender. Across diverse college samples (e.g., first-year students, graduating students), internal consistency reliability estimates were found acceptable, with values exceeding 0.80. Additionally, SOS scores have been reported to be (a) positively correlated with response time effort on a computer-based assessment, (b) minimally correlated to measures of quantitative and scientific reasoning, and (c) low, non-significant correlations to Scholastic Aptitude Test scores (Wise and Kong, 2005). Currently, less is known about the scale’s functioning when administered to high school students. Furthermore, it is unknown how the instrument’s psychometric properties function for ELLs. Based on the increasing use of assessments to drive accountability and evaluative decisions, combined with the increasingly diversity of students in the classroom, research is needed to determine the extent to which scores can be interpreted and used similarly to operationalize test-taking motivation across ELLs and non-ELLs. The appropriate use of self-report measures of test-taking motivation requires the accumulation of empirical evidence of their psychometric properties if they are to be used to build validity arguments for the substantive use of large-scale assessment results.

Measurement invariance (MI) is a desired statistical property of test scores that indicates their equivalence across diverse groups (e.g., gender, language; Meredith, 1993; Vandenberg and Lance, 2000). As such, it can be considered a necessary step to the interpretation and use of scores across different language, culture, or treatment groups. The utility of large-scale assessment scores among policy-makers, educators, and researchers is largely based on the implicit assumption that scores have met the property of MI. Consequently, depending on the purpose(s) of the test, a lack of MI may have serious consequences for test-users and examinees. Conversely, evidence of the MI of obtained scores across language groups indicates that scores have similar meaning across groups, thus supporting for score validity for intended purposes.

Multi-sample confirmatory factor analysis (MCFA) is a model-based approach to formally testing the MI of a scale’s factor structure (Vandenberg and Lance, 2000). Specifically, MCFA provides a basis to determine the extent to which measurement model parameters (e.g., factor loadings) are invariant, provided that the instrument’s theoretical factor structure reports acceptable model-data fit across groups (i.e., configural invariance; Kline, 2016). It is based on the comparison of increasingly restrictive factor analytic models that differ in terms of the model parameters specified as invariant across groups (Vandenberg and Lance, 2000). For ordered-categorical data, typical measurement model parameters tested for invariance include: factor loadings, thresholds, and error variances (Millsap and Tein, 2004). Meredith (1993) indicated that a scale’s measurement model can demonstrate three types of factorial invariance: weak, strong, and strict. Weak factorial invariance is present when the factor loadings indicating the strength of relationship between the observed and latent variables are invariant across groups. Strong factorial invariance requires the additional invariance of thresholds. Last, strict factorial invariance requires equal error variances in addition to invariant factor loadings and thresholds across groups. *Partial measurement invariance* occurs when equality holds for certain parameters of an instrument’s factor structure (Byrne et al., 1989). The degrees of MI indicate the extent to which scores can be considered construct valid across diverse groups. Specifically, a lack of MI suggests scores should be interpreted with caution. Toward this end, MCFA was used to formally test the measurement invariance of the theoretical two-factor structure of the SOS across ELLs and non-ELLs. Such evidence provides relevant information pertaining to the construct validity of SOS scores when based on diverse language groups. Study implications relate to the use of empirically based instruments to guide programmatic decision-making related to diverse students’ test-taking motivation.

The purpose of this study was to investigate the factorial invariance of the SOS across 10th grade ELLs and non-ELLs attending a large district in the California (CA) Central Valley. The context of the assessment of students’ test-taking motivation was in relation to completion of both a low- and high-stakes assessment. Specifically, the low-stakes assessment was an end-of-grade state standards assessment designed to measure grade 10 students’ attainment of grade standards. The high-stakes assessment was the California high school exit exam, a high-stakes assessment used to award students a high school diploma. In the U.S., where students designated as ELLs represent the fastest growing student population subgroup, substantiating validity claims for measures of test-taking motivation is paramount to inform K-12 educational policy and practices. The *Standards for Educational and Psychological Testing* (hereafter referred to as the *Standards*; American Educational Research Association, American Psychological Association, and National Council on Measurement in Education, 2014]) provide criteria to substantiate score use and interpretation. Study implications tie to the measurement of test-taking motivation of ELLs and non-ELLs in the context of low- and high-stakes assessments.

## Materials and Methods

### Participants

Data were based on 10th grade high school students in a large school district in the California (CA) Central Valley (*N* = 5,257; 48.1% female). Student demographics included: 5.8% African American; 4% Asian; 61.1% Latino/a; 0.6% Native American; 26.9% white; and, 1.4% other. ELLs comprised 41.8% of sample, whereas 60.5% of all students were eligible for free/reduced lunch and 2.8% qualified for special education. Parents’ educational level was: 22.6% not high school graduate; 26.2% high school graduate; 21.1% some college; 13.1% college graduate; and, 9.4% post graduate, respectively.

### Instrumentation

The SOS is a self-report 10-item measure of examinees’ test-taking motivation (Sundre, 2007). It is to be administered following the completion of a large-scale assessment and includes two subscales, Importance and Effort, each comprised of five items. Responses are provided on a Likert scale (i.e., *Strongly Disagree* to *Strongly Agree*) to yield two subscale scores to operationalize students’ test-taking motivation with high scores indicative of high levels of importance and effort associated with test engagement. Its two-factor theoretical structure is based on the expectancy-value model of achievement motivation (Pintrich, 1989; Wolf and Smith, 1995; Sundre, 1999). The instrument is to be used to characterize students’ general test-taking motivation, as opposed to guiding decisions about individual students.

Several investigations have been conducted examining the scale’s psychometric properties. Data based on college students have indicated that the internal consistency reliability estimates (i.e., Cronbach’s coefficient alpha) of subscales exceed 0.80 for use among low- and high-stakes assessments (Sundre, 2007). Factor analytic results have supported the scale’s two-factor structure (Sundre and Finney, 2002), with subscale reliabilities deemed acceptable (Sundre, 2007). Additional information is provided by Sundre (2007).

Within the present study, the SOS was administered by classroom teachers immediately following completion of the California (CA) low-stakes, end-of-grade standards assessment, or the CA Standards Test (CST; California Department of Education, 2011) and the high-stakes CA High School Exit Exam (CAHSEE; California Department of Education, 2012). The CST is the CA mandated end-of-grade test, developed by Educational Testing Service, to measure students’ attainment of grade level standards (e.g., ELA; California Department of Education, 2011). The CST is administered across grades 2–11, with scores indicative of students’ attainment of grade standards. The CAHSEE is administered to grade 10 students and is comprised of two parts: English Language Arts and mathematics. The SOS was administered in English and students recorded their answers using a scantron sheet. The study was carried out in accordance with the recommendations of the school district policy and approval by university IRB committee.

### Data Analysis

Item analyses were conducted to estimate descriptive statistics for the item- and scale-level data. Internal consistency reliability (Cronbach’s alpha) was used to examine subscale score consistency, with values above 0.80 desired (Henson, 2001).

Multi-sample confirmatory factor analysis was used to test the MI of the SOS across ELLs and non-ELLs (Cheung and Rensvold, 2002; Kline, 2016). Robust weighted least squares (WLSMV; Muthén et al., 1997, Unpublished) was used for parameter estimation using MPLUS 7.31 (Muthén et al., 1998–2015). Evaluation of mode-data fit included: chi-square statistic (WLSMV), root mean square error of approximation (*RMSEA*), and comparative fit index (*CFI*). *RMSEA* values less than 0.05 were used to indicate good model fit and those less than 0.08 suggested reasonable fit (Hu and Bentler, 1999). The *CFI* values above 0.95 were used to indicate acceptable fit (Hu and Bentler, 1999).

Sequential, nested model comparisons were used to formally test the MI of the SOS factor structure across language groups (Millsap and Tein, 2004). Based on configural invariance in which the scale’s theoretical model was found to be acceptable across groups, measurement parameters of focus included: factor loadings, thresholds, and residual variances. Criteria for lack of MI was based on a statistically significantly likelihood ratio chi-square difference statistic, χ^2^_Difference_. However, as the chi-square difference statistic (χ^2^_Difference_) is susceptible toward the rejection of the null hypothesis of equivalent model parameters in large sample sizes, an incremental change in the *CFI* of less than 0.01 between nested models also was used as criteria to accept model parameter invariance (Cheung and Rensvold, 2002; Chen, 2007). Provided a finding of MI, a follow-up latent means structure analysis was conducted to compare groups on the latent means (Hancock, 2004). The MPLUS procedures for invariance testing included the use of WLSMV (DIFFTEST), as well as the *theta parameterization* option to test error variance equality (Muthén et al., 1998–2015). The TYPE = COMPLEX command was used to account for the non-independence of students within schools for the estimation of standard errors and the test of model-data fit.

## Results

### Item Analysis

**Table 1** reports item-level descriptive statistics of the SOS for the low-stakes, end-of-grade assessment. Overall, language groups agreed with the importance and effort placed toward completion of the high school exit exam. In particular, Item 1 received the highest rating across groups (“Doing well on this test was important to me”). With the exception of Items 3 (“I am not curious about how I did on this test relative to others.”) and 7 (“While taking this test, I could have worked harder on it.”), item-total correlations were moderate. Internal consistency estimates fell below 0.80 across subscales. Therefore, item analysis results supported deletion of Items 3 and 7, which resulted in subscale score alpha estimates above 0.80 across samples.

**Table 1:** Descriptive statistics of SOS scale items across ELL and non-ELL^a^ samples for Low-Stakes Assessment.

	Mean	*SD*	Median	Range	Item-total correlation
**Importance**					
1	4.35 (4.43)	0.99 (0.92)	5 (5)	4 (4)	0.67 (0.65)
3	3.57 (3.59)	1.24 (1.19)	4 (4)	4 (4)	0.37 (0.34)
4	4.06 (4.06)	1.15 (1.14)	4 (4)	4 (4)	0.58 (0.66)
5	4.07 (4.20)	1.04 (0.98)	4(4)	4 (4)	0.66 (0.63)
8	4.17 (4.20)	1.05 (0.98)	4 (4)	4 (4)	0.68 (0.64)
**Effort**					
2	4.21 (4.18)	0.93 (0.90)	4 (5)	4 (4)	0.71 (0.67)
6	4.19 (4.15)	0.97 (0.94)	4 (4)	4 (4)	0.71 (0.67)
7	3.14 (2.78)	1.23 (1.23)	3 (3)	4 (4)	0.41 (0.25)
9	3.89 (3.86)	1.16 (1.12)	4 (4)	4 (4)	0.62 (0.58)
10	4.02 (3.88)	0.98 (0.90)	4 (4)	4 (4)	0.56 (0.51)


*^a^Values in parenthesis. SD, standard deviation.*

**Table 2** reports item-level descriptive statistics of the SOS for the high-stakes assessment (i.e., high school exit exam). Overall, language groups agreed with the importance and effort placed toward completion of the high school exit exam. Similar to previously reported results, item-total correlations were moderate, except for Items 3 (“I am not curious about how I did on this test relative to others”) and 7 (“While taking this test, I could have worked harder on it”) which were relatively low. Across language groups, Item 1 (“Doing well on this test was important to me”) received the highest average ratings. Internal consistency estimates were low across groups (e.g., <0.70 for Effort). Therefore, item analysis results supported deletion of Items 3 and 7, which resulted in subscale score alpha estimates above 0.75 across samples.

**Table 2:** Descriptive statistics of SOS scale items across ELL and non-ELL^a^ samples for High-Stakes Assessment.

	Mean	*SD*	Median	Range	Item-Total Correlation
**Importance**					
1	4.67 (4.63)	0.78 (0.80)	5 (5)	4 (4)	0.55 (0.57)
3	3.52 (3.51)	1.21 (1.24)	4 (4)	4 (4)	0.20 (0.22)
4	4.26 (4.29)	1.13 (1.12)	5 (5)	4 (4)	0.44 (0.44)
5	4.58 (4.47)	0.83 (0.90)	5 (5)	4 (4)	0.53 (0.55)
8	4.50 (4.47)	0.83 (0.88)	4 (4)	4 (4)	0.57 (0.59)
**Effort**					
2	4.35 (4.37)	0.81 (0.82)	4 (5)	4 (4)	0.57 (0.64)
6	4.47 (4.47)	0.81 (0.82)	5 (5)	4 (4)	0.55 (0.66)
7	2.79 (3.29)	1.18 (1.21)	3 (3)	4 (4)	0.25 (0.37)
9	4.19 (4.20)	1.03 (1.02)	4 (4)	4 (4)	0.51 (0.58)
10	4.02 (4.16)	0.87 (0.91)	4 (4)	4 (4)	0.41 (0.47)


*^a^Values in parenthesis. SD, standard deviation.*

### Measurement Invariance of SOS Factor Structure

#### Low-Stakes Assessment

The two-factor model was found to provide acceptable model-data fit for the entire sample, χ^2^ (38) = 732.87, *p* < 0.01, *RMSEA* = 0.08 (90% CIs = 0.07–0.09), and *CFI* = 0.99. It was also acceptable when fit to each group’s data separately. **Table 3** reports the model parameter estimates of factor loadings, structure coefficients, and error variances across groups. The matrix of factor loadings was found to be invariant, χ^2^_Difference_ (6) = 8.27, *p*_Difference_ = 0.22. The subsequent model with equality constraints on the thresholds resulted in a statistically significant decline in model-data fit based on chi-square value [χ^2^_Difference_ (22) = 52.55, *p*_Difference_ < 0.01]. However, the difference between the *CFI* values was less than criteria of 0.01 and, as a result, threshold invariance was deemed acceptable. A test of invariance of the residual variances reported a chi-square difference statistic that statistically significant at the 0.01 level [χ^2^_Difference_ (8) = 50.13, *p*_Difference_ = 0.04], but the incremental change in CFI was less than 0.01. Therefore, invariance of the residual invariances was found to be tenable. A follow-up comparison of latent means indicated similar levels of importance but that non-ELLs reported lower effort (-0.22), with a negligible effect size of 0.02.

**Table 3:** Final two-factor model factor loadings, structure coefficients, and error variances for ELLs and non-ELLs^a^ for Low-Stakes Assessment.

	Importance		Effort		Error variance
			
Items	*P*	*S*	*P*	*S*	
1^b^	0.94 (0.94)	0.94 (0.94)	–	0.84 (0.85)	0.12 (0.12)
4	0.66 (0.64)	0.66 (0.64)	–	0.58 (0.58)	0.15 (0.22)
5	0.87 (0.88)	0.87 (0.88)	–	0.77 (0.79)	0.57 (0.60)
8	0.84 (0.78)	0.84 (0.78)	–	0.75 (0.70)	0.25 (0.23)
2^b^	–	0.82 (0.74)	0.92 (0.88)	0.92 (0.88)	0.17 (0.17)
6	–	0.81 (0.77)	0.91 (0.91)	0.91 (0.91)	0.30 (0.39)
9	–	0.61 (0.57)	0.68 (0.67)	0.68 (0.67)	0.54 (0.53)
10	–	0.65 (0.56)	0.73 (0.66)	0.73 (0.66)	0.47 (0.55)


*Completely standardized solution reported. Pattern coefficients fixed to zero are indicated by a dash. All pattern coefficients were statistically significant (*p*s < 0.05). P, pattern coefficient; S, structure coefficient.*

*^a^Parameter estimates in parenthesis. ^b^Parameter set to 1.0 to set factor scales.*

#### High-Stakes Assessment

The two-factor model was found to provide acceptable model-data fit for the entire sample, χ^2^ (38) = 461.44, *p* < 0.01, *RMSEA* = 0.06 (90% CIs = 0.06 – 0.07), and *CFI* = 0.98. It was also acceptable when fit to each group’s data separately. **Table 4** reports the model parameter estimates of factor loadings and error variances across groups. The matrix of factor loadings was found to be invariant, χ^2^_Difference_ (6) = 12.44, *p*_Difference_ = 0.05. Subsequently, a model with the additional constraints of invariant thresholds resulted in a statistically significant decline in model-data fit based on chi-square value [χ^2^_Difference_ (22) = 75.23, *p*_Difference_ < 0.01], but inspection of the difference in *RMSEA* and *CFI* values was less than criteria of 0.01; thus, threshold invariance was deemed met. Last, although the chi-square difference statistic was statistically significant at the 0.05 level [χ^2^_Difference_ (8) = 15.88, *p*_Difference_ = 0.04], the incremental change in CFI was less than 0.01. Thus, residual invariance was found to be acceptable. A follow-up comparison of latent means indicated similar levels of importance but that non-ELLs reported lower effort (-0.15), with a negligible effect size of 0.09.

**Table 4:** Final two-factor model factor loadings, structure coefficients, and error variances for ELLs and non-ELLs^a^ for High-Stakes Assessment.

	Importance		Effort		Error variance
			
Items	*P*	*S*	*P*	*S*	
1^b^	0.91 (0.95)	0.91 (0.95)	–	0.81 (0.80)	0.17 (0.10)
4	0.55 (0.52)	0.55 (0.52)	–	0.49 (0.44)	0.69 (0.73)
5	0.87 (0.85)	0.87 (0.85)	–	0.77 (0.72)	0.25 (0.28)
8	0.74 (0.78)	0.74 (0.78)	–	0.66 (0.66)	0.45 (0.39)
2^b^	–	0.75 (0.74)	0.84 (0.87)	0.84 (0.87)	0.30 (0.24)
6	–	0.79 (0.77)	0.89 (0.91)	0.89 (0.91)	0.21 (0.17)
9	–	0.58 (0.57)	0.65 (0.67)	0.65 (0.67)	0.58 (0.55)
10	–	0.52 (0.56)	0.58 (0.66)	0.58 (0.66)	0.66 (0.56)


*Completely standardized solution reported. Pattern coefficients fixed to zero are indicated by a dash. All pattern coefficients were statistically significant (*p*s < 0.05). P, pattern coefficient; S, structure coefficient.*

*^a^Parameter estimates in parenthesis. ^b^Parameter set to 1.0 to set factor scales.*

**Table 5** reports descriptive statistics and internal consistency reliability (Cronbach’s alpha) for the Importance and Effort sub-scales for the low- and high-stakes assessments. As shown, the highest score that could be reported is 20 across assessments. Overall, student groups reported higher levels of agreement for the importance and effort put forth on the high-stakes assessment, compared to the low-stakes assessment. As shown, subscale score internal consistency estimates exceeded 0.75 across samples, with slightly higher values reported for the measure when administered with the low-stakes assessment.

**Table 5:** SOS descriptive information for ELLs and non-ELLs^a^ across Low- and High-Stakes Assessments.

Subscale/Scale	Mean	*SD*	α	
			
			ELLs	Non-ELLs
**Low-Stakes**				
Importance	16.65 (16.88)	3.45 (3.23)	0.83	0.81
Effort	16.29 (16.07)	3.30 (3.11)	0.83	0.81
**High-Stakes**				
Importance	17.85 (18.02)	2.84 (2.72)	0.75	0.76
Effort	17.18 (16.99)	2.81 (2.64)	0.75	0.80


*^a^Values in parenthesis unless otherwise specified.*

*Importance items: 1, 4, 5, and 8; Effort items: 2, 6, 9, and 10.*

*ELL, English language learners; non-ELLs, non-English language learners; SD, standard deviation; α, Cronbach’s coefficient alpha.*

## Discussion

Large-scale assessments play a prominent role in a range of decisions related to the evaluation of student and school outcomes (Davies, 2008). Student test-taking motivation has been reported as a determinate of test performance (Karmos and Karmos, 1984; O’Neil et al., 1995/1996; Wise and DeMars, 2005, 2010; Wise et al., 2006; Wise, 2009; Abdelfattah, 2010). For example, students may not be motivated to do well on a low-stakes assessment in which they are not held accountable for their performance (Wolf and Smith, 1995; Wolf et al., 1995). On the other hand, in the context of high-stakes assessments, it would be expected that students would be motivated to demonstrate their academic proficiency due to the consequences of poor test performance. When students’ motivation levels are low because they do not place value on their test performance, results from the test may underestimate students’ abilities and lead to inaccurate measures of a school or testing program’s effectiveness (Meijer and Sijtsma, 1995, 2001; DeMars, 2000; Napoli and Raymond, 2004; Putwain, 2008). Consequently, consideration of students’ test-taking motivation may be a critical factor in determining the validity and accuracy of large-scale assessment results. As such, in large-scale assessment settings, understanding examinee test-taking motivation may provide test users valuable information regarding the degree to which test performance may be impacted by motivational factors. This may be particularly relevant when seeking to interpret and use large-scale assessment results obtained from diverse examinees.

This study sought to address the gap in the literature regarding the measurement of ELL and non-ELL high school students’ test-taking motivation in the context of both low- and high-stakes assessments. The low-stakes assessment was the end-of-grade state standards assessment designed to determine students’ attainment of grade level standards, whereas the high-stake assessment was the CA high school exit exam, required for students to receive a high school diploma. In educational settings in which large-scale assessment results are used to guide policy, accountability, and evaluative decisions, among others, the construct validity of obtained scores is of critical importance. The selection of the SOS to assess grade 10 students’ test-taking motivation for the CA high school exit exam was based on the availability of existing research regarding the scale’s development and psychometric properties (e.g.,Sundre and Finney, 2002; Sundre, 2007). The SOS was developed to aid test users to determine the degree to which students were motivated to perform on a particular large-scale assessment. Existing literature has yielded favorable evidence on the scale’s psychometric properties across high school and college-aged samples. Notably, prior research has noted that instrument may be most applicable in the context of low-stakes assessments due to previous findings that motivation may be elevated for high-stakes assessments in which scores have direct consequences for examinees (Sundre, 2007). While informative, no information has been provided to-date on its functioning or psychometric properties when administered to diverse student language groups in high school.

Therefore, the MI of the SOS two-factor structure was empirically tested across grade 10 ELL and non-ELL students. With the proliferation of ELLs in the educational system, educators are seeking strategies to best address their learning needs, the availability of psychometrically sound measures for programmatic use is both timely and necessary. In the broader context of this study, the persistent academic disparities in test performance across ELLs and non-ELLs lead to administrators seeking empirical evidence on the extent to which test-taking motivation may be a factor to consider in how students approach the diverse assessments. In contrast to previous studies (Sundre, 2007), item analysis results supported the removal of one item from each of the Importance (Item 3) and Effort (Item 7) subscales, which resulted in increased reliability prior to testing the scale’s MI. Specifically, Item 3 dealt with an examinee’s curiosity of test performance relative to others, whereas Item 7 inquired into whether an examinee could have worked harder on the assessment. In relation to Importance subscale questions, Item 3 is the only item that asks examinees about their curiosity of their normative test performance. In comparison to other items comprising the Effort subscale, Item 7 asks examinees if they could have worked harder on the assessment instead of their degree of effort on the completed assessment. In light of previous investigations of the psychometric properties of the SOS based on older high school and college-aged students, continued research is needed to determine the grades and ages in which the scale may be expected to yield reliable scores.

Multi-sample confirmatory factor analysis results reported that the parameters comprising the SOS two-factor structure were invariant across language groups in the context of both low- and high-stakes assessments. Specifically, factor loading invariance suggested that the relationship between items and factors (i.e., Importance, Effort) were the same across groups. Similarly, threshold invariance indicated that the location on the underlying latent continuum (test-taking motivation) in which an examinee would have a higher probability of selecting a particular categorical response is the same across groups. Lastly, the finding of residual invariance suggests that variance unaccounted for by the importance and effort factors is the same across groups. As a result, the instrument demonstrated strict factorial invariance (Meredith, 1993) for low- and high-stakes assessments. A follow-up comparison of latent means indicated that non-ELLs reported a slightly lower effort, whereas both groups attached similar levels of importance to the assessments. Thus, within this study, ELLs and non-ELLs have similar levels of test-taking motivation. As such, the modified version of the SOS in this study based on the removal of Items 3 and 7 was found to function similarly across ELLs and non-ELLs. Such evidence supports the meaning of scores across groups and, thus, the construct validity of SOS scores. The collection of validity evidence pertaining to the functioning of scores across diverse language groups is in accordance to the *Standards* (American Educational Research Association, American Psychological Association, and National Council on Measurement in Education, 2014), particularly regarding the interpretation and use of large-scale assessment results.

While the scale’s two-factor structure was found to be invariant across groups, continued research on the measurement of students’ test-taking motivation is recommended. That is, the removal of two items was found to improve the internal consistency of scale scores, thus reducing the degree to which subscale item sets may provide adequate coverage of the measured traits of importance and effort. Second, the generalizability of findings is needed to identify the extent to which SOS scores may be used across other grade levels. Whereas the instrument was developed and validated on an older student population (Sundre, 2007), this study represents a step toward generalizing the psychometric properties of scores among diverse student sub-groups in lower grades. Indeed, factor analytic studies are needed to establish the extent to which obtained scores may be construct valid for other student sub-groups (e.g., gender). The present findings also support the premise that the instrument may be more useful for assessing test-taking motivation in low-stakes assessment context. This is due to the fact that the instrument’s scores were very high and had a much more restrictive range for the high-stakes assessment, a consideration identified by Sundre (2007). Thus, more fully understanding students’ test-taking motivation in the context of high-stakes assessments is an area of future research. In addition, empirical evidence regarding the predictive utility of scores of test-taking motivation measures is needed to inform test users of the degree to which student non-cognitive beliefs are determinants of large-scale test performance. The collection of validity evidence on obtained scores provides key information pertaining to the use of non-cognitive assessment scores for decision-making purposes for interpreting large-scale assessment results.

The heterogeneity of students in the U.S. educational system requires consideration of the extent to which instruments designed to guide practical, research, and policy decisions function across diverse students. In the context of large-scale assessments, test users need assurance that obtained results truly reflect examinees’ achievement and are psychometrically sound. Evidence of this nature is increasingly important as educational reform efforts continually tie accountability decisions to student performance on large-scale assessments (e.g., end-of-grade). Toward this end, this study provides critical information pertaining to the functioning of one theoretically based measure of students’ test-taking motivation. Whereas the instrument was developed and validated on upper high school and college students, less was known regarding its functioning among different high school grade levels. Toward this end, results supported the invariance of the instrument’s two-factor structure across ELL and non-ELL samples. Nonetheless, additional research is needed to determine the generalizability of this finding across diverse contexts and populations.

## Author Contributions

JI was responsible for all aspects of writing, data analysis, and reporting. In collaboration with DM, he reviewed literature, designed study, and conducted analyses. DM was responsible for all aspects of section of measures and data collection. DM also contributed to the writing of the manuscript.

## Conflict of Interest Statement

The authors declare that the research was conducted in the absence of any commercial or financial relationships that could be construed as a potential conflict of interest.
